# (2,3,5,10,12,13,15,20-Octa­phenyl­porphinato)copper(II) 1,1,2,2-tetra­chloro­ethane solvate

**DOI:** 10.1107/S1600536808000147

**Published:** 2008-01-09

**Authors:** Puttaiah Bhyrappa, Karuppaiah Karunanithi, Babu Varghese

**Affiliations:** a Department of Chemistry, Indian Institute of Technology Madras, Chennai 600 036, India; b Sophisticated Analytical Instrument Facility, Indian Institute of Technology Madras, Chennai 600 036, India

## Abstract

The title complex, [Cu(C_68_H_44_N_4_)]·C_2_H_2_Cl_4_, exhibits nearly square-planar geometry around the Cu^II^ centre and the macrocyclic ring is almost planar. The porphyrin mol­ecule has an approximate non-crystallographic inversion centre (*C_i_*), and a non-crystallographic twofold rotation axis (*C*_2_) within the Cu^II^–porphyrin ring plane. Further, it has non-crystallographic twofold rotation axis and mirror plane (*C_s_*) symmetry perpendicular to the mol­ecular plane. The mol­ecular packing of the complexes and the solvent molecules shows weak inter­molecular C—H⋯π, C—H⋯Cl and C—H⋯N inter­actions, forming a clathrate-like structure.

## Related literature

For related structures, see: Chan *et al.* (1994 ▶); Fleischer *et al.* (1964 ▶). For porphyrin sponges, see: Byrn *et al.* (1993 ▶). For the preparation of the CuTPP(Ph)_4_ complex, see: Bhyrappa *et al.* (2006 ▶); Adler *et al.* (1970 ▶). For hydrogen-bonding inter­actions, see: Desiraju & Steiner (1999 ▶); Steiner (2002 ▶).
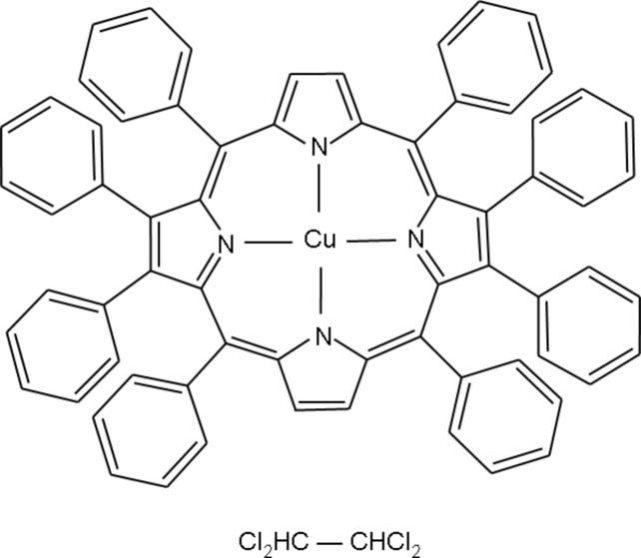


## Experimental

### 

#### Crystal data

[Cu(C_68_H_44_N_4_)]·C_2_H_2_Cl_4_
*M* *_r_* = 1148.45
Monoclinic, 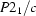

*a* = 18.8891 (5) Å
*b* = 12.2800 (4) Å
*c* = 24.5323 (7) Å
β = 106.239 (1)°
*V* = 5463.4 (3) Å^3^
*Z* = 4
Mo *K*α radiation
μ = 0.65 mm^−1^
*T* = 173 (2) K
0.28 × 0.24 × 0.20 mm

#### Data collection

Bruker APEXII CCD area-detector diffractometer
Absorption correction: multi-scan (*SADABS*; Bruker 2003 ▶) *T*_min_ = 0.840, *T*_max_ = 0.882
33284 measured reflections
9594 independent reflections
6243 reflections with *I* > 2σ(*I*)
*R*_int_ = 0.042

#### Refinement

*R*[*F* ^2^ > 2σ(*F* ^2^)] = 0.045
*wR*(*F* ^2^) = 0.118
*S* = 1.00
9594 reflections
712 parameters
H-atom parameters constrained
Δρ_max_ = 0.68 e Å^−3^
Δρ_min_ = −0.55 e Å^−3^

### 

Data collection: *APEX2* (Bruker, 2004 ▶); cell refinement: *APEX2* and *SAINT-Plus* (Bruker, 2004 ▶); data reduction: *SAINT-Plus* and *XPREP* (Bruker, 2003 ▶); program(s) used to solve structure: *SHELXS97* (Sheldrick, 2008 ▶); program(s) used to refine structure: *SHELXL97* (Sheldrick, 2008 ▶); molecular graphics: *ORTEP-3* (Farrugia, 1997 ▶), *WinGX* (Farrugia, 1999 ▶) and *Mercury* (Macrae *et al.*, 2006 ▶); software used to prepare material for publication: *SHELXL97*.

## Supplementary Material

Crystal structure: contains datablocks global, I. DOI: 10.1107/S1600536808000147/si2064sup1.cif

Structure factors: contains datablocks I. DOI: 10.1107/S1600536808000147/si2064Isup2.hkl

Additional supplementary materials:  crystallographic information; 3D view; checkCIF report

## Figures and Tables

**Table 1:** Hydrogen-bond geometry (Å, °)

*D*—H⋯*A*	*D*—H	H⋯*A*	*D*⋯*A*	*D*—H⋯*A*
C70—H70⋯N1^i^	0.98	2.46	3.430 (5)	170
C58—H58⋯Cl1^i^	0.93	2.91	3.728 (5)	148
C66—H66⋯C18^i^	0.93	2.83	3.755 (4)	172
C67—H67⋯Cl4	0.93	2.93	3.424 (4)	115
C34—H34⋯C6^ii^	0.93	2.90	3.790 (5)	161
C35—H35⋯C11^ii^	0.93	2.89	3.821 (5)	175
C41—H41⋯C19^ii^	0.93	2.89	3.725 (5)	149

Symmetry codes: (i) 
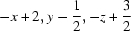
; (ii) 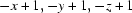.

**Fig. 1.**
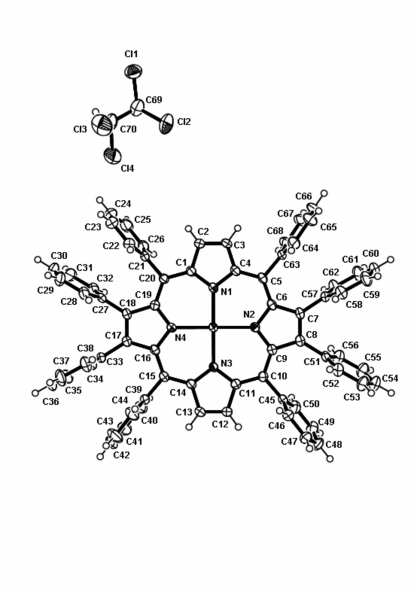
ORTEP plot of the molecular structure of CuTPP(Ph)4.TCE. Thermal ellipsoids shown at the 50% probability level.

**Fig. 2.**
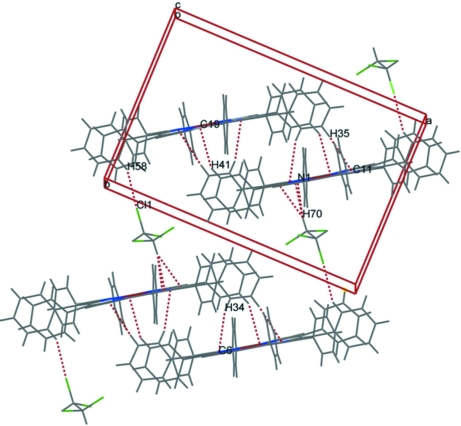
Molecular packing diagram of the CuTPP(Ph)4.TCE complex with a view along the unit cell c axis. Color scheme: C, H, gray; N, blue; Cl, green; Cu, red. The dotted red lines show the intermolecular C—H···π (H34···C6, H41···C19, H58···Cl1) and C—H···N (H70···N1) interactions.

**Fig. 3.**
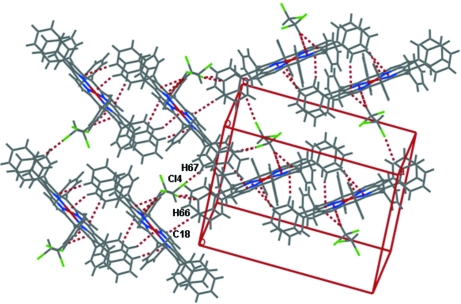
View of the molecular packing of interconnecting two arrays to form a two-dimensional network of the title structure. Color scheme: C, H, gray; N, blue; Cl, green; Cu, red. The dotted red lines show the inter-array hydrogen bonding C—H···Cl (H67···Cl4) and C—H···π (H66···C18) interactions.

## supplementary crystallographic information

### Comment

The title complex shows a non-crystallographic centre of inversion (C_i_) at the Cu^II^ centre, a rotational axis, (C_2_) in plane of the porphyrin ring and another C_2_ axis and a mirror plane (C_s_) perpendicular to the molecular plane. The Cu^II^ centre has nearly square planar geometry (Fig. 1) and it is quite similar to the 5,10,15,20-tetraphenylporphinato copper(II) complex, CuTPP structure (Fleischer *et al.*, 1964). The crystal structure of H_2_TPP(Ph)_4_ exhibited planar conformation of the porphyrin ring (Chan *et al.*, 1994). In the title complex, shortening of the Cu—N distance (1.961 (2) Å) was observed along the β-pyrrole without substituents relative to the distance (2.060 (2) Å) towards other β-pyrroles with phenyl groups. The average Cu—N bond length was found to be 2.010 (2)Å and it is longer than that observed for the corresponding CuTPP (1.981 (7) Å) (Fleischer *et al.*, 1964) complex. The mean plane deviation of the atoms from the 24-atom core indicates the near planar geometry of the porphyrin ring with a maximum displacement of the core atoms (0.077 (7) Å). The Cu^II^ ion deviates from the 24-atom core by 0.0167 (6) Å. The phenyl groups at the *meso*-positions and β-pyrrole carbons are oriented perpendicular to the mean plane of the porphyrin ring with dihedral angles of 81.1 (3)° and 80.3 (5)°, respectively. The *meso*-carbon-to-phenyl distance (C—C = 1.502 (4) Å) was found to be marginally longer than the β-pyrrole carbon-to-phenyl distance (C—C = 1.494 (4) Å) indicating minimal conjugation of the phenyl rings with the porphyrin π-system.

The molecular packing of a porphyrin array oriented approximately along the *c* axis is shown in Fig.2. The porphyrins form a slipped stack dimers and the porphyrin ring planes are separated by 4.791 Å. The dimers are held together through three symmetry related C—H···π interactions in the range 2.89 - 2.90 Å (Table 1). These long distances indicate weak C—H···π interactions (Steiner, 2002). The non-covalently bonded dimeric units are bridged by solvate mediated hydrogen bonding interactions. Each array is interconnected *via* interporphyrin, weak C—H···π (H66···C18 = 2.83 Å) and porphyrin-solvate hydrogen bonding (H67···Cl4 = 2.93 Å) interactions (Fig.3 and Table 1). The close contact distances are expected for the presence of hydrogen bonding (C—H···Cl and C—H···N) interactions (Desiraju & Steiner, 1999). The porphyrin ring planes in the nearest adjacent array are oriented at a skewed angle of 66.6° to each other. Each such two-dimensional network stack along the unit cell *c* axis by solvate mediated intermolecular interactions. Similar solvate encapsulated porphyrin sponges have been reported in the literature (Byrn *et al.*, 1993).

### Experimental

2,3,12,13-Tetraphenyl-5,10,15,20-tetraphenylporphyrin, H_2_TPP(Ph)_4_, was prepared using the variation of the reported procedure (Bhyrappa *et al.*, 2006) by employing excess of super base (40 mmol) and the reaction was completed in 15 h with 92% yield of the H_2_TPP(Ph)_4_ derivative. Its CuTPP(Ph)_4_ complex was prepared using a literature method (Adler *et al.*, 1970). The crystals of CuTPP(Ph)_4_ were grown by direct diffusion of cyclohexane into 1,1,2,2-tetrachloroethane solution of the porphyrin over a period of three days.

### Refinement

All the H atoms were placed in constrained positions (C—H = 0.93- 0.98 Å) and refined using a riding model with *U*_iso_(H) = 1.2 or 1.5 times *U*_eq_(C) on the parent atom.

### Crystal data

**Table:**

[Cu(C_68_H_44_N_4_)]·C_2_H_2_Cl_4_	*F*_000_ = 2364
*M**_r_* = 1148.45	*D*_x_ = 1.396 Mg m^−^^3^
Monoclinic, *P*2_1_/*c*	Mo *K*α radiation λ = 0.71073 Å
Hall symbol: -P 2ybc	Cell parameters from 6786 reflections
*a* = 18.8891 (5) Å	θ = 2.4–26.1º
*b* = 12.2800 (4) Å	µ = 0.65 mm^−^^1^
*c* = 24.5323 (7) Å	*T* = 173 (2) K
β = 106.239 (1)º	Plate, brown
*V* = 5463.4 (3) Å^3^	0.28 × 0.24 × 0.20 mm
*Z* = 4	

### Data collection

**Table:**

Bruker APEXII CCD area-detector diffractometer	9594 independent reflections
Radiation source: fine-focus sealed tube	6243 reflections with *I* > 2σ(*I*)
Monochromator: graphite	*R*_int_ = 0.042
*T* = 173(2) K	θ_max_ = 25.0º
ω and φ scans	θ_min_ = 2.3º
Absorption correction: multi-scan(SADABS; Bruker 2003)	*h* = −22→16
*T*_min_ = 0.840, *T*_max_ = 0.882	*k* = −14→14
33284 measured reflections	*l* = −29→28

### Refinement

**Table:**

Refinement on *F*^2^	Secondary atom site location: difference Fourier map
Least-squares matrix: full	Hydrogen site location: inferred from neighbouring sites
*R*[*F*^2^ > 2σ(*F*^2^)] = 0.046	H-atom parameters constrained
*wR*(*F*^2^) = 0.119	*w* = 1/[σ^2^(*F*_o_^2^) + (0.0464*P*)^2^ + 5.7309*P*] where *P* = (*F*_o_^2^ + 2*F*_c_^2^)/3
*S* = 1.00	(Δ/σ)_max_ < 0.001
9594 reflections	Δρ_max_ = 0.68 e Å^−^^3^
712 parameters	Δρ_min_ = −0.55 e Å^−^^3^
Primary atom site location: structure-invariant direct methods	Extinction correction: none

### Special details

**Table:**

Geometry. All e.s.d.'s (except the e.s.d. in the dihedral angle between two l.s. planes) are estimated using the full covariance matrix. The cell e.s.d.'s are taken into account individually in the estimation of e.s.d.'s in distances, angles and torsion angles; correlations between e.s.d.'s in cell parameters are only used when they are defined by crystal symmetry. An approximate (isotropic) treatment of cell e.s.d.'s is used for estimating e.s.d.'s involving l.s. planes.
Refinement. Refinement of *F*^2^ against ALL reflections. The weighted *R*-factor *wR* and goodness of fit *S* are based on *F*^2^, conventional *R*-factors *R* are based on *F*, with *F* set to zero for negative *F*^2^. The threshold expression of *F*^2^ > σ(*F*^2^) is used only for calculating *R*-factors(gt) *etc*. and is not relevant to the choice of reflections for refinement. *R*-factors based on *F*^2^ are statistically about twice as large as those based on *F*, and *R*- factors based on ALL data will be even larger.

### Fractional atomic coordinates and isotropic or equivalent isotropic displacement parameters (Å^2^)

**Table:**

	*x*	*y*	*z*	*U*_iso_*/*U*_eq_	
C1	0.64459 (17)	0.5928 (2)	0.62180 (13)	0.0231 (7)	
C2	0.57857 (17)	0.6535 (3)	0.62036 (13)	0.0248 (7)	
C3	0.54667 (17)	0.6822 (3)	0.56593 (13)	0.0239 (7)	
C4	0.59271 (16)	0.6397 (2)	0.53228 (13)	0.0222 (7)	
C5	0.58044 (16)	0.6525 (3)	0.47372 (13)	0.0236 (7)	
C6	0.62698 (17)	0.6117 (3)	0.44359 (13)	0.0241 (7)	
C7	0.61224 (18)	0.6229 (3)	0.38328 (13)	0.0304 (8)	
H7	0.5727	0.6599	0.3593	0.036*	
C8	0.66522 (18)	0.5709 (3)	0.36775 (14)	0.0319 (8)	
H8	0.6695	0.5650	0.3310	0.038*	
C9	0.71477 (17)	0.5255 (3)	0.41811 (13)	0.0241 (7)	
C10	0.77607 (17)	0.4639 (3)	0.41720 (13)	0.0238 (7)	
C11	0.82656 (17)	0.4216 (2)	0.46542 (13)	0.0225 (7)	
C12	0.89196 (16)	0.3591 (3)	0.46627 (13)	0.0240 (7)	
C13	0.92241 (17)	0.3272 (3)	0.52072 (13)	0.0249 (7)	
C14	0.87621 (16)	0.3701 (3)	0.55416 (13)	0.0228 (7)	
C15	0.88581 (16)	0.3516 (3)	0.61190 (12)	0.0239 (7)	
C16	0.84064 (17)	0.3967 (3)	0.64239 (13)	0.0246 (7)	
C17	0.85496 (18)	0.3817 (3)	0.70261 (13)	0.0303 (8)	
H17	0.8932	0.3413	0.7261	0.036*	
C18	0.80283 (17)	0.4369 (3)	0.71861 (13)	0.0292 (8)	
H18	0.7984	0.4419	0.7553	0.035*	
C19	0.75556 (17)	0.4864 (3)	0.66928 (12)	0.0247 (7)	
C20	0.69373 (17)	0.5476 (3)	0.66969 (13)	0.0231 (7)	
C21	0.68139 (17)	0.5585 (3)	0.72752 (13)	0.0288 (8)	
C22	0.6517 (2)	0.4722 (3)	0.75038 (15)	0.0429 (10)	
H22	0.6351	0.4108	0.7283	0.052*	
C23	0.6464 (2)	0.4758 (4)	0.80535 (16)	0.0590 (13)	
H23	0.6267	0.4173	0.8203	0.071*	
C24	0.6702 (2)	0.5662 (5)	0.83754 (17)	0.0659 (14)	
H24	0.6663	0.5692	0.8745	0.079*	
C25	0.6998 (2)	0.6523 (4)	0.81635 (17)	0.0604 (13)	
H25	0.7160	0.7134	0.8388	0.073*	
C26	0.7058 (2)	0.6487 (3)	0.76105 (15)	0.0423 (9)	
H26	0.7263	0.7072	0.7467	0.051*	
C27	0.54803 (17)	0.6852 (3)	0.66816 (13)	0.0259 (7)	
C28	0.5045 (2)	0.6149 (3)	0.68860 (15)	0.0406 (9)	
H28	0.4969	0.5443	0.6745	0.049*	
C29	0.4719 (2)	0.6481 (3)	0.72986 (16)	0.0483 (10)	
H29	0.4425	0.5998	0.7430	0.058*	
C30	0.4827 (2)	0.7509 (4)	0.75122 (16)	0.0463 (10)	
H30	0.4617	0.7725	0.7795	0.056*	
C31	0.5249 (2)	0.8226 (3)	0.73069 (16)	0.0476 (10)	
H31	0.5313	0.8935	0.7444	0.057*	
C32	0.55786 (19)	0.7895 (3)	0.68968 (14)	0.0372 (9)	
H32	0.5870	0.8382	0.6765	0.045*	
C33	0.47542 (16)	0.7434 (3)	0.54840 (13)	0.0250 (7)	
C34	0.41086 (18)	0.6902 (3)	0.54866 (15)	0.0368 (9)	
H34	0.4123	0.6167	0.5580	0.044*	
C35	0.34450 (19)	0.7451 (3)	0.53527 (16)	0.0425 (10)	
H35	0.3016	0.7083	0.5357	0.051*	
C36	0.3414 (2)	0.8534 (3)	0.52138 (16)	0.0439 (10)	
H36	0.2966	0.8902	0.5119	0.053*	
C37	0.4057 (2)	0.9076 (3)	0.52158 (16)	0.0411 (9)	
H37	0.4041	0.9811	0.5123	0.049*	
C38	0.47228 (18)	0.8533 (3)	0.53543 (14)	0.0320 (8)	
H38	0.5153	0.8907	0.5361	0.038*	
C39	0.51471 (17)	0.7143 (3)	0.43896 (12)	0.0251 (7)	
C40	0.44515 (18)	0.6684 (3)	0.42313 (14)	0.0345 (8)	
H40	0.4380	0.5985	0.4352	0.041*	
C41	0.38612 (19)	0.7259 (3)	0.38938 (16)	0.0449 (10)	
H41	0.3394	0.6947	0.3791	0.054*	
C42	0.3960 (2)	0.8287 (3)	0.37089 (15)	0.0454 (10)	
H42	0.3559	0.8673	0.3485	0.054*	
C43	0.4649 (2)	0.8745 (3)	0.38543 (14)	0.0401 (9)	
H43	0.4719	0.9438	0.3726	0.048*	
C44	0.52382 (18)	0.8173 (3)	0.41928 (13)	0.0307 (8)	
H44	0.5705	0.8486	0.4290	0.037*	
C45	0.78249 (17)	0.4373 (3)	0.35884 (13)	0.0274 (8)	
C46	0.7451 (2)	0.3482 (3)	0.33074 (14)	0.0404 (9)	
H46	0.7181	0.3043	0.3485	0.048*	
C47	0.7477 (2)	0.3239 (4)	0.27625 (16)	0.0524 (11)	
H47	0.7224	0.2636	0.2575	0.063*	
C48	0.7869 (2)	0.3877 (4)	0.24988 (16)	0.0536 (12)	
H48	0.7890	0.3705	0.2134	0.064*	
C49	0.8231 (2)	0.4771 (4)	0.27717 (16)	0.0586 (13)	
H49	0.8495	0.5212	0.2590	0.070*	
C50	0.8210 (2)	0.5028 (3)	0.33185 (15)	0.0433 (10)	
H50	0.8455	0.5641	0.3501	0.052*	
C51	0.92391 (17)	0.3269 (3)	0.41925 (13)	0.0267 (8)	
C52	0.90578 (19)	0.2281 (3)	0.39251 (14)	0.0363 (9)	
H52	0.8705	0.1844	0.4016	0.044*	
C53	0.9393 (2)	0.1929 (3)	0.35218 (15)	0.0483 (10)	
H53	0.9266	0.1257	0.3347	0.058*	
C54	0.9907 (2)	0.2558 (4)	0.33792 (16)	0.0497 (11)	
H54	1.0122	0.2327	0.3101	0.060*	
C55	1.0106 (2)	0.3541 (4)	0.36498 (18)	0.0538 (11)	
H55	1.0463	0.3968	0.3559	0.065*	
C56	0.9775 (2)	0.3896 (3)	0.40592 (16)	0.0428 (10)	
H56	0.9914	0.4557	0.4243	0.051*	
C57	0.99055 (17)	0.2602 (3)	0.53830 (12)	0.0257 (7)	
C58	0.98709 (19)	0.1484 (3)	0.54570 (14)	0.0337 (8)	
H58	0.9415	0.1150	0.5406	0.040*	
C59	1.0503 (2)	0.0863 (3)	0.56045 (15)	0.0459 (10)	
H59	1.0472	0.0116	0.5656	0.055*	
C60	1.1178 (2)	0.1343 (4)	0.56752 (16)	0.0513 (11)	
H60	1.1604	0.0924	0.5782	0.062*	
C61	1.1226 (2)	0.2441 (4)	0.55881 (16)	0.0465 (10)	
H61	1.1683	0.2764	0.5629	0.056*	
C62	1.05912 (18)	0.3068 (3)	0.54386 (14)	0.0349 (9)	
H62	1.0625	0.3810	0.5375	0.042*	
C63	0.94523 (17)	0.2782 (3)	0.64525 (13)	0.0279 (8)	
C64	0.9268 (2)	0.1744 (3)	0.65906 (14)	0.0379 (9)	
H64	0.8782	0.1506	0.6465	0.045*	
C65	0.9809 (3)	0.1058 (3)	0.69174 (16)	0.0529 (11)	
H65	0.9683	0.0365	0.7012	0.064*	
C66	1.0524 (3)	0.1401 (4)	0.70990 (16)	0.0569 (12)	
H66	1.0886	0.0937	0.7312	0.068*	
C67	1.0710 (2)	0.2428 (4)	0.69686 (15)	0.0497 (11)	
H67	1.1197	0.2659	0.7093	0.060*	
C68	1.01747 (18)	0.3120 (3)	0.66534 (14)	0.0375 (9)	
H68	1.0303	0.3823	0.6575	0.045*	
N1	0.65247 (13)	0.5851 (2)	0.56727 (10)	0.0215 (6)	
N2	0.69068 (13)	0.5520 (2)	0.46464 (10)	0.0221 (6)	
N3	0.81909 (13)	0.4296 (2)	0.51955 (10)	0.0214 (6)	
N4	0.77967 (13)	0.4618 (2)	0.62234 (10)	0.0225 (6)	
Cu1	0.735277 (19)	0.50618 (3)	0.543368 (15)	0.02048 (11)	
C69	1.2045 (2)	0.3335 (4)	0.94084 (18)	0.0544 (11)	
H69	1.1761	0.2674	0.9422	0.065*	
C70	1.2450 (2)	0.3183 (4)	0.89730 (19)	0.0594 (12)	
H70	1.2792	0.2574	0.9098	0.071*	
Cl1	1.14390 (8)	0.44408 (13)	0.92542 (6)	0.0953 (5)	
Cl2	1.27110 (6)	0.35061 (10)	1.00777 (4)	0.0613 (3)	
Cl3	1.29698 (8)	0.43045 (13)	0.88925 (6)	0.0882 (4)	
Cl4	1.17999 (7)	0.27836 (13)	0.83291 (5)	0.0837 (4)	

### Atomic displacement parameters (Å^2^)

**Table:**

	*U*^11^	*U*^22^	*U*^33^	*U*^12^	*U*^13^	*U*^23^
C1	0.0263 (17)	0.0209 (18)	0.0243 (18)	−0.0048 (14)	0.0110 (14)	−0.0054 (14)
C2	0.0265 (17)	0.0230 (18)	0.0284 (18)	−0.0018 (15)	0.0137 (14)	−0.0052 (14)
C3	0.0261 (17)	0.0186 (17)	0.0294 (18)	−0.0026 (14)	0.0116 (14)	−0.0026 (14)
C4	0.0230 (17)	0.0185 (17)	0.0265 (18)	−0.0039 (14)	0.0094 (14)	−0.0025 (14)
C5	0.0241 (17)	0.0211 (18)	0.0263 (18)	−0.0002 (14)	0.0083 (14)	−0.0007 (14)
C6	0.0263 (17)	0.0204 (18)	0.0259 (18)	−0.0002 (14)	0.0080 (14)	0.0007 (14)
C7	0.0329 (19)	0.036 (2)	0.0224 (18)	0.0102 (16)	0.0082 (15)	0.0037 (15)
C8	0.037 (2)	0.039 (2)	0.0223 (18)	0.0076 (17)	0.0124 (15)	0.0033 (16)
C9	0.0285 (18)	0.0235 (19)	0.0219 (17)	0.0007 (14)	0.0097 (14)	−0.0010 (14)
C10	0.0279 (18)	0.0227 (18)	0.0232 (17)	−0.0011 (14)	0.0112 (14)	−0.0011 (14)
C11	0.0261 (17)	0.0202 (18)	0.0242 (17)	−0.0038 (14)	0.0121 (14)	−0.0005 (14)
C12	0.0238 (17)	0.0251 (18)	0.0249 (18)	−0.0011 (14)	0.0096 (13)	−0.0017 (14)
C13	0.0261 (17)	0.0258 (19)	0.0267 (18)	−0.0005 (14)	0.0135 (14)	−0.0015 (15)
C14	0.0215 (16)	0.0216 (18)	0.0250 (17)	−0.0015 (14)	0.0061 (13)	−0.0016 (14)
C15	0.0234 (17)	0.0266 (19)	0.0219 (17)	−0.0007 (14)	0.0067 (13)	−0.0020 (14)
C16	0.0248 (17)	0.0276 (19)	0.0225 (17)	−0.0007 (15)	0.0084 (14)	−0.0013 (14)
C17	0.0311 (19)	0.039 (2)	0.0205 (17)	0.0071 (16)	0.0070 (14)	0.0022 (15)
C18	0.0317 (19)	0.040 (2)	0.0175 (17)	0.0025 (16)	0.0090 (14)	0.0004 (15)
C19	0.0270 (17)	0.0277 (19)	0.0210 (16)	−0.0028 (15)	0.0093 (13)	−0.0051 (14)
C20	0.0267 (18)	0.0221 (18)	0.0233 (17)	−0.0053 (14)	0.0114 (14)	−0.0066 (14)
C21	0.0262 (18)	0.041 (2)	0.0190 (17)	0.0105 (16)	0.0064 (14)	−0.0046 (16)
C22	0.042 (2)	0.060 (3)	0.031 (2)	−0.006 (2)	0.0174 (17)	−0.0002 (19)
C23	0.050 (3)	0.102 (4)	0.031 (2)	0.000 (3)	0.0195 (19)	0.013 (2)
C24	0.054 (3)	0.124 (5)	0.022 (2)	0.021 (3)	0.015 (2)	−0.004 (3)
C25	0.067 (3)	0.078 (4)	0.030 (2)	0.015 (3)	0.005 (2)	−0.026 (2)
C26	0.046 (2)	0.045 (2)	0.033 (2)	0.0071 (19)	0.0071 (17)	−0.0125 (18)
C27	0.0250 (17)	0.029 (2)	0.0247 (17)	0.0053 (15)	0.0093 (14)	−0.0014 (15)
C28	0.046 (2)	0.038 (2)	0.045 (2)	−0.0014 (19)	0.0259 (19)	−0.0092 (18)
C29	0.050 (2)	0.055 (3)	0.052 (3)	−0.001 (2)	0.034 (2)	0.001 (2)
C30	0.047 (2)	0.062 (3)	0.036 (2)	0.019 (2)	0.0207 (19)	−0.001 (2)
C31	0.062 (3)	0.041 (2)	0.045 (2)	0.009 (2)	0.023 (2)	−0.013 (2)
C32	0.043 (2)	0.037 (2)	0.036 (2)	−0.0014 (18)	0.0178 (17)	−0.0053 (17)
C33	0.0254 (17)	0.0271 (19)	0.0246 (17)	0.0019 (15)	0.0103 (14)	−0.0051 (15)
C34	0.033 (2)	0.027 (2)	0.052 (2)	−0.0004 (17)	0.0166 (17)	0.0025 (17)
C35	0.027 (2)	0.041 (2)	0.061 (3)	−0.0019 (18)	0.0156 (18)	0.005 (2)
C36	0.030 (2)	0.041 (2)	0.062 (3)	0.0104 (19)	0.0158 (18)	0.002 (2)
C37	0.041 (2)	0.026 (2)	0.058 (3)	0.0073 (18)	0.0156 (18)	0.0039 (18)
C38	0.0309 (19)	0.026 (2)	0.042 (2)	−0.0018 (16)	0.0156 (16)	−0.0013 (16)
C39	0.0279 (18)	0.0271 (19)	0.0211 (17)	0.0035 (15)	0.0083 (13)	−0.0036 (14)
C40	0.032 (2)	0.034 (2)	0.037 (2)	−0.0023 (17)	0.0088 (16)	−0.0093 (17)
C41	0.026 (2)	0.052 (3)	0.050 (2)	0.0013 (19)	−0.0010 (17)	−0.019 (2)
C42	0.038 (2)	0.053 (3)	0.038 (2)	0.023 (2)	−0.0027 (17)	−0.006 (2)
C43	0.050 (2)	0.035 (2)	0.035 (2)	0.0145 (19)	0.0111 (18)	0.0052 (17)
C44	0.0305 (19)	0.033 (2)	0.0285 (18)	0.0026 (16)	0.0085 (15)	−0.0001 (16)
C45	0.0266 (18)	0.033 (2)	0.0224 (17)	0.0071 (16)	0.0068 (14)	−0.0018 (15)
C46	0.050 (2)	0.042 (2)	0.031 (2)	0.0024 (19)	0.0141 (17)	−0.0029 (18)
C47	0.058 (3)	0.059 (3)	0.036 (2)	0.006 (2)	0.007 (2)	−0.017 (2)
C48	0.048 (2)	0.090 (4)	0.026 (2)	0.012 (2)	0.0156 (19)	−0.010 (2)
C49	0.056 (3)	0.093 (4)	0.034 (2)	−0.013 (3)	0.026 (2)	−0.003 (2)
C50	0.043 (2)	0.059 (3)	0.032 (2)	−0.012 (2)	0.0178 (17)	−0.005 (2)
C51	0.0292 (18)	0.030 (2)	0.0234 (17)	0.0087 (15)	0.0108 (14)	0.0010 (15)
C52	0.040 (2)	0.041 (2)	0.031 (2)	0.0006 (18)	0.0164 (16)	−0.0023 (17)
C53	0.059 (3)	0.053 (3)	0.036 (2)	0.008 (2)	0.017 (2)	−0.014 (2)
C54	0.052 (3)	0.067 (3)	0.038 (2)	0.021 (2)	0.0258 (19)	−0.001 (2)
C55	0.052 (3)	0.063 (3)	0.064 (3)	0.006 (2)	0.043 (2)	0.008 (2)
C56	0.050 (2)	0.039 (2)	0.051 (2)	0.0011 (19)	0.032 (2)	−0.0015 (19)
C57	0.0291 (18)	0.032 (2)	0.0183 (16)	0.0046 (16)	0.0099 (13)	−0.0019 (14)
C58	0.037 (2)	0.035 (2)	0.033 (2)	0.0061 (17)	0.0148 (16)	−0.0009 (16)
C59	0.061 (3)	0.036 (2)	0.042 (2)	0.018 (2)	0.016 (2)	0.0043 (18)
C60	0.042 (2)	0.064 (3)	0.046 (2)	0.030 (2)	0.0095 (19)	0.000 (2)
C61	0.027 (2)	0.064 (3)	0.049 (2)	0.008 (2)	0.0123 (17)	−0.007 (2)
C62	0.034 (2)	0.038 (2)	0.036 (2)	0.0015 (17)	0.0146 (16)	−0.0065 (17)
C63	0.0302 (19)	0.034 (2)	0.0225 (17)	0.0088 (16)	0.0118 (14)	0.0005 (15)
C64	0.043 (2)	0.038 (2)	0.038 (2)	0.0074 (18)	0.0202 (17)	0.0028 (18)
C65	0.080 (3)	0.044 (3)	0.044 (2)	0.025 (2)	0.033 (2)	0.016 (2)
C66	0.063 (3)	0.074 (3)	0.034 (2)	0.041 (3)	0.014 (2)	0.012 (2)
C67	0.039 (2)	0.072 (3)	0.035 (2)	0.016 (2)	0.0065 (18)	0.002 (2)
C68	0.034 (2)	0.048 (2)	0.0300 (19)	0.0074 (18)	0.0084 (16)	0.0007 (18)
N1	0.0235 (14)	0.0203 (15)	0.0214 (14)	−0.0013 (12)	0.0074 (11)	−0.0012 (11)
N2	0.0251 (14)	0.0216 (14)	0.0221 (14)	0.0019 (12)	0.0105 (11)	0.0003 (12)
N3	0.0236 (14)	0.0231 (15)	0.0186 (14)	−0.0007 (12)	0.0077 (11)	−0.0002 (11)
N4	0.0249 (14)	0.0227 (15)	0.0212 (14)	−0.0010 (12)	0.0087 (11)	−0.0028 (11)
Cu1	0.0220 (2)	0.0212 (2)	0.01977 (19)	0.00008 (17)	0.00838 (14)	−0.00128 (16)
C69	0.045 (2)	0.050 (3)	0.070 (3)	0.008 (2)	0.019 (2)	0.009 (2)
C70	0.051 (3)	0.050 (3)	0.080 (3)	0.016 (2)	0.023 (2)	0.009 (2)
Cl1	0.0875 (10)	0.1020 (11)	0.0990 (11)	0.0603 (9)	0.0303 (8)	0.0086 (9)
Cl2	0.0466 (6)	0.0845 (9)	0.0481 (6)	−0.0065 (6)	0.0054 (5)	0.0052 (6)
Cl3	0.0867 (10)	0.0987 (11)	0.0909 (10)	−0.0276 (8)	0.0437 (8)	0.0083 (8)
Cl4	0.0745 (8)	0.1147 (12)	0.0581 (8)	0.0044 (8)	0.0122 (6)	−0.0277 (7)

### Geometric parameters (Å, °)

**Table:**

C1—N1	1.390 (4)	C37—C38	1.379 (5)
C1—C20	1.393 (4)	C37—H37	0.93
C1—C2	1.445 (4)	C38—H38	0.93
C2—C3	1.349 (4)	C39—C44	1.381 (5)
C2—C27	1.496 (4)	C39—C40	1.382 (4)
C3—C4	1.453 (4)	C40—C41	1.383 (5)
C3—C33	1.496 (4)	C40—H40	0.93
C4—N1	1.385 (4)	C41—C42	1.371 (5)
C4—C5	1.398 (4)	C41—H41	0.93
C5—C6	1.390 (4)	C42—C43	1.370 (5)
C5—C39	1.501 (4)	C42—H42	0.93
C6—N2	1.380 (4)	C43—C44	1.381 (5)
C6—C7	1.434 (4)	C43—H43	0.93
C7—C8	1.329 (4)	C44—H44	0.93
C7—H7	0.93	C45—C50	1.372 (5)
C8—C9	1.438 (4)	C45—C46	1.378 (5)
C8—H8	0.93	C46—C47	1.384 (5)
C9—N2	1.381 (4)	C46—H46	0.93
C9—C10	1.388 (4)	C47—C48	1.359 (6)
C10—C11	1.395 (4)	C47—H47	0.93
C10—C45	1.506 (4)	C48—C49	1.366 (6)
C11—N3	1.378 (4)	C48—H48	0.93
C11—C12	1.450 (4)	C49—C50	1.390 (5)
C12—C13	1.356 (4)	C49—H49	0.93
C12—C51	1.497 (4)	C50—H50	0.93
C13—C14	1.453 (4)	C51—C52	1.375 (5)
C13—C57	1.486 (4)	C51—C56	1.382 (5)
C14—N3	1.381 (4)	C52—C53	1.385 (5)
C14—C15	1.396 (4)	C52—H52	0.93
C15—C16	1.397 (4)	C53—C54	1.360 (6)
C15—C63	1.492 (4)	C53—H53	0.93
C16—N4	1.375 (4)	C54—C55	1.378 (6)
C16—C17	1.437 (4)	C54—H54	0.93
C17—C18	1.341 (4)	C55—C56	1.394 (5)
C17—H17	0.93	C55—H55	0.93
C18—C19	1.424 (4)	C56—H56	0.93
C18—H18	0.93	C57—C62	1.387 (4)
C19—N4	1.385 (4)	C57—C58	1.389 (5)
C19—C20	1.392 (4)	C58—C59	1.377 (5)
C20—C21	1.507 (4)	C58—H58	0.93
C21—C26	1.380 (5)	C59—C60	1.371 (6)
C21—C22	1.388 (5)	C59—H59	0.93
C22—C23	1.381 (5)	C60—C61	1.372 (6)
C22—H22	0.93	C60—H60	0.93
C23—C24	1.363 (7)	C61—C62	1.385 (5)
C23—H23	0.93	C61—H61	0.93
C24—C25	1.365 (7)	C62—H62	0.93
C24—H24	0.93	C63—C68	1.379 (5)
C25—C26	1.393 (5)	C63—C64	1.388 (5)
C25—H25	0.93	C64—C65	1.392 (5)
C26—H26	0.93	C64—H64	0.93
C27—C32	1.378 (5)	C65—C66	1.365 (6)
C27—C28	1.379 (5)	C65—H65	0.93
C28—C29	1.385 (5)	C66—C67	1.371 (6)
C28—H28	0.93	C66—H66	0.93
C29—C30	1.361 (5)	C67—C68	1.380 (5)
C29—H29	0.93	C67—H67	0.93
C30—C31	1.374 (5)	C68—H68	0.93
C30—H30	0.93	N1—Cu1	2.060 (2)
C31—C32	1.384 (5)	N2—Cu1	1.961 (2)
C31—H31	0.93	N3—Cu1	2.061 (2)
C32—H32	0.93	N4—Cu1	1.961 (2)
C33—C38	1.384 (4)	C69—C70	1.488 (6)
C33—C34	1.385 (4)	C69—Cl1	1.748 (4)
C34—C35	1.379 (5)	C69—Cl2	1.779 (4)
C34—H34	0.93	C69—H69	0.98
C35—C36	1.371 (5)	C70—Cl3	1.734 (5)
C35—H35	0.93	C70—Cl4	1.777 (5)
C36—C37	1.383 (5)	C70—H70	0.98
C36—H36	0.93		
			
N1—C1—C20	124.0 (3)	C39—C40—C41	120.2 (4)
N1—C1—C2	109.6 (3)	C39—C40—H40	119.9
C20—C1—C2	126.4 (3)	C41—C40—H40	119.9
C3—C2—C1	107.6 (3)	C42—C41—C40	120.5 (3)
C3—C2—C27	123.0 (3)	C42—C41—H41	119.8
C1—C2—C27	129.4 (3)	C40—C41—H41	119.8
C2—C3—C4	107.2 (3)	C43—C42—C41	119.9 (3)
C2—C3—C33	122.2 (3)	C43—C42—H42	120.1
C4—C3—C33	130.6 (3)	C41—C42—H42	120.1
N1—C4—C5	124.3 (3)	C42—C43—C44	119.7 (4)
N1—C4—C3	109.6 (3)	C42—C43—H43	120.2
C5—C4—C3	126.1 (3)	C44—C43—H43	120.2
C6—C5—C4	123.9 (3)	C43—C44—C39	121.2 (3)
C6—C5—C39	115.2 (3)	C43—C44—H44	119.4
C4—C5—C39	120.9 (3)	C39—C44—H44	119.4
N2—C6—C5	127.4 (3)	C50—C45—C46	119.4 (3)
N2—C6—C7	109.5 (3)	C50—C45—C10	121.7 (3)
C5—C6—C7	123.0 (3)	C46—C45—C10	118.8 (3)
C8—C7—C6	107.8 (3)	C45—C46—C47	120.2 (4)
C8—C7—H7	126.1	C45—C46—H46	119.9
C6—C7—H7	126.1	C47—C46—H46	119.9
C7—C8—C9	107.6 (3)	C48—C47—C46	120.4 (4)
C7—C8—H8	126.2	C48—C47—H47	119.8
C9—C8—H8	126.2	C46—C47—H47	119.8
N2—C9—C10	127.7 (3)	C47—C48—C49	119.7 (4)
N2—C9—C8	109.4 (3)	C47—C48—H48	120.2
C10—C9—C8	122.9 (3)	C49—C48—H48	120.2
C9—C10—C11	124.4 (3)	C48—C49—C50	120.6 (4)
C9—C10—C45	115.0 (3)	C48—C49—H49	119.7
C11—C10—C45	120.5 (3)	C50—C49—H49	119.7
N3—C11—C10	124.1 (3)	C45—C50—C49	119.7 (4)
N3—C11—C12	109.9 (3)	C45—C50—H50	120.2
C10—C11—C12	125.9 (3)	C49—C50—H50	120.2
C13—C12—C11	107.0 (3)	C52—C51—C56	118.7 (3)
C13—C12—C51	122.1 (3)	C52—C51—C12	120.0 (3)
C11—C12—C51	130.9 (3)	C56—C51—C12	121.0 (3)
C12—C13—C14	107.2 (3)	C51—C52—C53	120.9 (3)
C12—C13—C57	122.7 (3)	C51—C52—H52	119.6
C14—C13—C57	130.2 (3)	C53—C52—H52	119.6
N3—C14—C15	124.6 (3)	C54—C53—C52	120.5 (4)
N3—C14—C13	109.5 (3)	C54—C53—H53	119.7
C15—C14—C13	125.8 (3)	C52—C53—H53	119.7
C14—C15—C16	123.6 (3)	C53—C54—C55	119.5 (3)
C14—C15—C63	121.3 (3)	C53—C54—H54	120.3
C16—C15—C63	115.1 (3)	C55—C54—H54	120.3
N4—C16—C15	127.9 (3)	C54—C55—C56	120.3 (4)
N4—C16—C17	109.9 (3)	C54—C55—H55	119.9
C15—C16—C17	122.1 (3)	C56—C55—H55	119.9
C18—C17—C16	107.1 (3)	C51—C56—C55	120.1 (4)
C18—C17—H17	126.5	C51—C56—H56	119.9
C16—C17—H17	126.5	C55—C56—H56	119.9
C17—C18—C19	107.7 (3)	C62—C57—C58	118.1 (3)
C17—C18—H18	126.1	C62—C57—C13	120.6 (3)
C19—C18—H18	126.1	C58—C57—C13	121.1 (3)
N4—C19—C20	126.6 (3)	C59—C58—C57	120.8 (3)
N4—C19—C18	109.9 (3)	C59—C58—H58	119.6
C20—C19—C18	123.6 (3)	C57—C58—H58	119.6
C19—C20—C1	124.9 (3)	C60—C59—C58	120.1 (4)
C19—C20—C21	113.9 (3)	C60—C59—H59	119.9
C1—C20—C21	121.1 (3)	C58—C59—H59	119.9
C26—C21—C22	118.5 (3)	C59—C60—C61	120.2 (4)
C26—C21—C20	121.2 (3)	C59—C60—H60	119.9
C22—C21—C20	120.0 (3)	C61—C60—H60	119.9
C23—C22—C21	121.2 (4)	C60—C61—C62	119.9 (4)
C23—C22—H22	119.4	C60—C61—H61	120.1
C21—C22—H22	119.4	C62—C61—H61	120.1
C24—C23—C22	119.3 (4)	C61—C62—C57	120.8 (4)
C24—C23—H23	120.3	C61—C62—H62	119.6
C22—C23—H23	120.3	C57—C62—H62	119.6
C23—C24—C25	120.9 (4)	C68—C63—C64	118.7 (3)
C23—C24—H24	119.5	C68—C63—C15	122.1 (3)
C25—C24—H24	119.5	C64—C63—C15	119.1 (3)
C24—C25—C26	120.0 (4)	C63—C64—C65	120.2 (4)
C24—C25—H25	120.0	C63—C64—H64	119.9
C26—C25—H25	120.0	C65—C64—H64	119.9
C21—C26—C25	120.1 (4)	C66—C65—C64	120.1 (4)
C21—C26—H26	120.0	C66—C65—H65	120.0
C25—C26—H26	120.0	C64—C65—H65	120.0
C32—C27—C28	118.2 (3)	C65—C66—C67	120.2 (4)
C32—C27—C2	120.1 (3)	C65—C66—H66	119.9
C28—C27—C2	121.5 (3)	C67—C66—H66	119.9
C27—C28—C29	120.9 (4)	C66—C67—C68	120.2 (4)
C27—C28—H28	119.5	C66—C67—H67	119.9
C29—C28—H28	119.5	C68—C67—H67	119.9
C30—C29—C28	120.3 (4)	C63—C68—C67	120.7 (4)
C30—C29—H29	119.8	C63—C68—H68	119.6
C28—C29—H29	119.8	C67—C68—H68	119.6
C29—C30—C31	119.5 (3)	C4—N1—C1	106.0 (2)
C29—C30—H30	120.2	C4—N1—Cu1	127.11 (19)
C31—C30—H30	120.2	C1—N1—Cu1	126.9 (2)
C30—C31—C32	120.2 (4)	C6—N2—C9	105.7 (2)
C30—C31—H31	119.9	C6—N2—Cu1	127.60 (19)
C32—C31—H31	119.9	C9—N2—Cu1	126.6 (2)
C27—C32—C31	120.7 (3)	C11—N3—C14	106.4 (2)
C27—C32—H32	119.6	C11—N3—Cu1	126.84 (19)
C31—C32—H32	119.6	C14—N3—Cu1	126.67 (19)
C38—C33—C34	118.6 (3)	C16—N4—C19	105.5 (2)
C38—C33—C3	122.2 (3)	C16—N4—Cu1	126.8 (2)
C34—C33—C3	119.0 (3)	C19—N4—Cu1	127.7 (2)
C35—C34—C33	120.8 (3)	N4—Cu1—N2	179.44 (11)
C35—C34—H34	119.6	N4—Cu1—N1	89.91 (10)
C33—C34—H34	119.6	N2—Cu1—N1	89.67 (10)
C36—C35—C34	120.4 (3)	N4—Cu1—N3	90.18 (10)
C36—C35—H35	119.8	N2—Cu1—N3	90.23 (10)
C34—C35—H35	119.8	N1—Cu1—N3	179.05 (10)
C35—C36—C37	119.3 (3)	C70—C69—Cl1	112.3 (3)
C35—C36—H36	120.4	C70—C69—Cl2	107.8 (3)
C37—C36—H36	120.4	Cl1—C69—Cl2	111.0 (2)
C38—C37—C36	120.5 (3)	C70—C69—H69	108.6
C38—C37—H37	119.7	Cl1—C69—H69	108.6
C36—C37—H37	119.7	Cl2—C69—H69	108.6
C37—C38—C33	120.4 (3)	C69—C70—Cl3	113.8 (3)
C37—C38—H38	119.8	C69—C70—Cl4	107.9 (3)
C33—C38—H38	119.8	Cl3—C70—Cl4	113.0 (3)
C44—C39—C40	118.5 (3)	C69—C70—H70	107.3
C44—C39—C5	119.8 (3)	Cl3—C70—H70	107.3
C40—C39—C5	121.6 (3)	Cl4—C70—H70	107.3
			
N1—C1—C2—C3	0.1 (4)	C5—C39—C40—C41	−177.7 (3)
C20—C1—C2—C3	−179.7 (3)	C39—C40—C41—C42	0.5 (5)
N1—C1—C2—C27	−178.4 (3)	C40—C41—C42—C43	0.6 (5)
C20—C1—C2—C27	1.9 (5)	C41—C42—C43—C44	−0.9 (5)
C1—C2—C3—C4	−0.1 (4)	C42—C43—C44—C39	−0.1 (5)
C27—C2—C3—C4	178.5 (3)	C40—C39—C44—C43	1.2 (5)
C1—C2—C3—C33	178.0 (3)	C5—C39—C44—C43	177.5 (3)
C27—C2—C3—C33	−3.4 (5)	C9—C10—C45—C50	91.7 (4)
C2—C3—C4—N1	0.1 (4)	C11—C10—C45—C50	−92.5 (4)
C33—C3—C4—N1	−177.8 (3)	C9—C10—C45—C46	−84.5 (4)
C2—C3—C4—C5	−178.9 (3)	C11—C10—C45—C46	91.3 (4)
C33—C3—C4—C5	3.1 (5)	C50—C45—C46—C47	1.3 (5)
N1—C4—C5—C6	−0.1 (5)	C10—C45—C46—C47	177.6 (3)
C3—C4—C5—C6	178.8 (3)	C45—C46—C47—C48	−0.1 (6)
N1—C4—C5—C39	−179.1 (3)	C46—C47—C48—C49	−0.9 (6)
C3—C4—C5—C39	−0.3 (5)	C47—C48—C49—C50	0.7 (7)
C4—C5—C6—N2	1.2 (5)	C46—C45—C50—C49	−1.5 (6)
C39—C5—C6—N2	−179.7 (3)	C10—C45—C50—C49	−177.7 (3)
C4—C5—C6—C7	178.1 (3)	C48—C49—C50—C45	0.5 (6)
C39—C5—C6—C7	−2.8 (5)	C13—C12—C51—C52	84.5 (4)
N2—C6—C7—C8	0.3 (4)	C11—C12—C51—C52	−93.1 (4)
C5—C6—C7—C8	−177.1 (3)	C13—C12—C51—C56	−88.7 (4)
C6—C7—C8—C9	0.1 (4)	C11—C12—C51—C56	93.7 (4)
C7—C8—C9—N2	−0.4 (4)	C56—C51—C52—C53	−1.3 (5)
C7—C8—C9—C10	178.9 (3)	C12—C51—C52—C53	−174.7 (3)
N2—C9—C10—C11	−3.0 (5)	C51—C52—C53—C54	−0.4 (6)
C8—C9—C10—C11	177.8 (3)	C52—C53—C54—C55	1.6 (6)
N2—C9—C10—C45	172.6 (3)	C53—C54—C55—C56	−1.1 (6)
C8—C9—C10—C45	−6.5 (5)	C52—C51—C56—C55	1.7 (5)
C9—C10—C11—N3	4.5 (5)	C12—C51—C56—C55	175.0 (3)
C45—C10—C11—N3	−170.9 (3)	C54—C55—C56—C51	−0.5 (6)
C9—C10—C11—C12	−178.6 (3)	C12—C13—C57—C62	76.4 (4)
C45—C10—C11—C12	6.0 (5)	C14—C13—C57—C62	−103.9 (4)
N3—C11—C12—C13	1.6 (4)	C12—C13—C57—C58	−99.0 (4)
C10—C11—C12—C13	−175.6 (3)	C14—C13—C57—C58	80.7 (4)
N3—C11—C12—C51	179.5 (3)	C62—C57—C58—C59	2.6 (5)
C10—C11—C12—C51	2.3 (5)	C13—C57—C58—C59	178.2 (3)
C11—C12—C13—C14	−0.2 (4)	C57—C58—C59—C60	−0.6 (5)
C51—C12—C13—C14	−178.3 (3)	C58—C59—C60—C61	−1.3 (6)
C11—C12—C13—C57	179.5 (3)	C59—C60—C61—C62	1.2 (6)
C51—C12—C13—C57	1.5 (5)	C60—C61—C62—C57	0.9 (5)
C12—C13—C14—N3	−1.3 (4)	C58—C57—C62—C61	−2.8 (5)
C57—C13—C14—N3	179.0 (3)	C13—C57—C62—C61	−178.3 (3)
C12—C13—C14—C15	176.6 (3)	C14—C15—C63—C68	79.8 (4)
C57—C13—C14—C15	−3.2 (6)	C16—C15—C63—C68	−101.7 (4)
N3—C14—C15—C16	−3.8 (5)	C14—C15—C63—C64	−103.5 (4)
C13—C14—C15—C16	178.6 (3)	C16—C15—C63—C64	74.9 (4)
N3—C14—C15—C63	174.5 (3)	C68—C63—C64—C65	−1.1 (5)
C13—C14—C15—C63	−3.0 (5)	C15—C63—C64—C65	−177.9 (3)
C14—C15—C16—N4	2.2 (5)	C63—C64—C65—C66	−0.6 (5)
C63—C15—C16—N4	−176.2 (3)	C64—C65—C66—C67	1.1 (6)
C14—C15—C16—C17	−175.8 (3)	C65—C66—C67—C68	0.0 (6)
C63—C15—C16—C17	5.7 (5)	C64—C63—C68—C67	2.3 (5)
N4—C16—C17—C18	0.3 (4)	C15—C63—C68—C67	178.9 (3)
C15—C16—C17—C18	178.7 (3)	C66—C67—C68—C63	−1.8 (5)
C16—C17—C18—C19	0.2 (4)	C5—C4—N1—C1	179.0 (3)
C17—C18—C19—N4	−0.6 (4)	C3—C4—N1—C1	−0.1 (3)
C17—C18—C19—C20	178.0 (3)	C5—C4—N1—Cu1	−1.9 (4)
N4—C19—C20—C1	1.4 (5)	C3—C4—N1—Cu1	179.00 (19)
C18—C19—C20—C1	−176.9 (3)	C20—C1—N1—C4	179.8 (3)
N4—C19—C20—C21	179.0 (3)	C2—C1—N1—C4	0.0 (3)
C18—C19—C20—C21	0.7 (5)	C20—C1—N1—Cu1	0.7 (4)
N1—C1—C20—C19	−2.0 (5)	C2—C1—N1—Cu1	−179.1 (2)
C2—C1—C20—C19	177.7 (3)	C5—C6—N2—C9	176.7 (3)
N1—C1—C20—C21	−179.5 (3)	C7—C6—N2—C9	−0.6 (3)
C2—C1—C20—C21	0.2 (5)	C5—C6—N2—Cu1	0.0 (5)
C19—C20—C21—C26	96.4 (4)	C7—C6—N2—Cu1	−177.3 (2)
C1—C20—C21—C26	−85.9 (4)	C10—C9—N2—C6	−178.6 (3)
C19—C20—C21—C22	−77.0 (4)	C8—C9—N2—C6	0.6 (3)
C1—C20—C21—C22	100.7 (4)	C10—C9—N2—Cu1	−1.9 (5)
C26—C21—C22—C23	0.1 (5)	C8—C9—N2—Cu1	177.4 (2)
C20—C21—C22—C23	173.7 (3)	C10—C11—N3—C14	174.9 (3)
C21—C22—C23—C24	0.5 (6)	C12—C11—N3—C14	−2.4 (3)
C22—C23—C24—C25	−0.6 (7)	C10—C11—N3—Cu1	−1.3 (4)
C23—C24—C25—C26	0.1 (7)	C12—C11—N3—Cu1	−178.6 (2)
C22—C21—C26—C25	−0.6 (5)	C15—C14—N3—C11	−175.6 (3)
C20—C21—C26—C25	−174.1 (3)	C13—C14—N3—C11	2.3 (3)
C24—C25—C26—C21	0.5 (6)	C15—C14—N3—Cu1	0.5 (4)
C3—C2—C27—C32	−77.1 (4)	C13—C14—N3—Cu1	178.4 (2)
C1—C2—C27—C32	101.1 (4)	C15—C16—N4—C19	−178.9 (3)
C3—C2—C27—C28	97.4 (4)	C17—C16—N4—C19	−0.6 (3)
C1—C2—C27—C28	−84.4 (4)	C15—C16—N4—Cu1	2.7 (5)
C32—C27—C28—C29	−0.4 (5)	C17—C16—N4—Cu1	−179.0 (2)
C2—C27—C28—C29	−175.0 (3)	C20—C19—N4—C16	−177.8 (3)
C27—C28—C29—C30	−0.3 (6)	C18—C19—N4—C16	0.7 (3)
C28—C29—C30—C31	1.4 (6)	C20—C19—N4—Cu1	0.6 (5)
C29—C30—C31—C32	−1.8 (6)	C18—C19—N4—Cu1	179.1 (2)
C28—C27—C32—C31	0.0 (5)	C16—N4—Cu1—N1	176.7 (3)
C2—C27—C32—C31	174.7 (3)	C19—N4—Cu1—N1	−1.4 (3)
C30—C31—C32—C27	1.1 (6)	C16—N4—Cu1—N3	−4.2 (3)
C2—C3—C33—C38	104.3 (4)	C19—N4—Cu1—N3	177.7 (3)
C4—C3—C33—C38	−78.0 (5)	C6—N2—Cu1—N1	−1.3 (3)
C2—C3—C33—C34	−71.4 (4)	C9—N2—Cu1—N1	−177.3 (3)
C4—C3—C33—C34	106.3 (4)	C6—N2—Cu1—N3	179.7 (3)
C38—C33—C34—C35	1.2 (5)	C9—N2—Cu1—N3	3.6 (3)
C3—C33—C34—C35	177.0 (3)	C4—N1—Cu1—N4	−178.1 (2)
C33—C34—C35—C36	0.1 (6)	C1—N1—Cu1—N4	0.8 (2)
C34—C35—C36—C37	−0.8 (6)	C4—N1—Cu1—N2	2.2 (2)
C35—C36—C37—C38	0.2 (6)	C1—N1—Cu1—N2	−178.9 (2)
C36—C37—C38—C33	1.1 (5)	C11—N3—Cu1—N4	178.2 (2)
C34—C33—C38—C37	−1.7 (5)	C14—N3—Cu1—N4	2.8 (3)
C3—C33—C38—C37	−177.5 (3)	C11—N3—Cu1—N2	−2.2 (2)
C6—C5—C39—C44	−73.2 (4)	C14—N3—Cu1—N2	−177.6 (3)
C4—C5—C39—C44	106.0 (4)	Cl1—C69—C70—Cl3	59.0 (4)
C6—C5—C39—C40	103.0 (4)	Cl2—C69—C70—Cl3	−63.6 (4)
C4—C5—C39—C40	−77.8 (4)	Cl1—C69—C70—Cl4	−67.2 (4)
C44—C39—C40—C41	−1.4 (5)	Cl2—C69—C70—Cl4	170.3 (2)

### Hydrogen-bond geometry (Å, °)

**Table:**

*D*—H···*A*	*D*—H	H···*A*	*D*···*A*	*D*—H···*A*
C70—H70···N1^i^	0.98	2.46	3.430 (5)	170
C58—H58···Cl1^i^	0.93	2.91	3.728 (5)	148
C66—H66···C18^i^	0.93	2.83	3.755 (4)	172
C67—H67···Cl4	0.93	2.93	3.424 (4)	115
C34—H34···C6^ii^	0.93	2.90	3.790 (5)	161
C35—H35···C11^ii^	0.93	2.89	3.821 (5)	175
C41—H41···C19^ii^	0.93	2.89	3.725 (5)	149

Symmetry codes: (i) −*x*+2, *y*−1/2, −*z*+3/2;  (ii) −*x*+1, −*y*+1, −*z*+1.
